# Prenatal diagnosis of mosaic chromosomal aneuploidy and uniparental disomy and clinical outcomes evaluation of four fetuses

**DOI:** 10.1186/s13039-023-00667-9

**Published:** 2023-12-06

**Authors:** Shengfang Qin, Xueyan Wang, Jin Wang, Na Xi, Mengjia Yan, Yuxia He, Mengling Ye, Zhuo Zhang, Yan Yin

**Affiliations:** Department of Medical Genetics and Prenatal Diagnosis, Sichuan Provincial Maternity and Child Health Care Hospital, Chengdu, 610045 Sichuan China

**Keywords:** Chromosome aneuploidy, Uniparental disomy, Mosaic, Prenatal diagnosis, Methylation, Clinical outcome

## Abstract

**Background:**

Few co-occurrence cases of mosaic aneuploidy and uniparental disomy (UPD) chromosomes have been reported in prenatal periods. It is a big challenge for us to predict fetal clinical outcomes with these chromosome abnormalities because of their highly heterogeneous clinical manifestations and limited phenotype attainable by ultrasound.

**Methods:**

Amniotic fluid samples were collected from four cases. Karyotype, chromosome microarray analysis, short tandem repeats, and whole exome sequencing were adopted to analyze fetal chromosomal aneuploidy, UPD, and gene variation. Meanwhile, CNVseq analysis proceeded for cultured and uncultured amniocytes in case 2 and case 4 and MS-MLPA for chr11 and chr15 in case 3.

**Results:**

All four fetuses showed mosaic chromosomal aneuploidy and UPD simultaneously. The results were: Case 1: T2(7%) and UPD(2)mat(12%). Case 2: T15(60%) and UPD(15)mat(40%). Case 3: 45,X(13%) and genome-wide paternal UPD(20%). Case 4: <10% of T20 and > 90% UPD(20)mat in uncultured amniocyte. By analyzing their formation mechanism of mosaic chromosomal aneuploidy and UPD, at least two adverse genetic events happened during their meiosis and mitosis. The fetus of case 1 presented a benign with a normal intrauterine phenotype, consistent with a low proportion of trisomy cells. However, the other three fetuses had adverse pregnancy outcomes, resulting from the UPD chromosomes with imprinted regions involved or a higher level of mosaic aneuploidy.

**Conclusion:**

UPD is often present with mosaic aneuploidy. It is necessary to analyze them simultaneously using a whole battery of analyses for these cases when their chromosomes with imprinted regions are involved or known carriers of a recessive allele. Fetal clinical outcomes were related to the affected chromosomes aneuploidy and UPD, mosaic levels and tissues, methylation status, and homozygous variation of recessive genes on the UPD chromosome. Genetic counseling for pregnant women with such fetuses is crucial to make informed choices.

## Background

Chromosomal aneuploidy is a common chromosomal abnormality, accounting for about 50% of the causes of spontaneous abortion of fetuses [1]. Complete aneuploidy is lethal, except for trisomies 13, 18, and 21, which can survive to term [2]. However, mosaic aneuploidies are found in 0.5% of live births and 0.1–0.3% in fetal amniotic fluid [3, 4]. Mosaic aneuploidies result from two principal mechanisms: postzygotic chromosome segregation errors and postzygotic trisomy/monosomy rescue of a pre-existing aneuploidy of meiotic origin. The clinical effects of chromosomal mosaicism are related to the size of the gene imbalance, the timing of the initial event, and the distribution of the abnormal cells in tissues [5, 6].

Uniparental disomy (UPD) refers to two homologous chromosomes inherited from the same parent. UPD is another chromosomal disorder with an incidence of 1.65/10,000 [7, 8]. Phenotypic effects from UPD are definite for several chromosomes, including 6pat, 7mat, 11pat, 14mat, 15mat, 15pat, and 20pat [9]. A mosaic chromosome may be related to the formation of UPD [10, 11]. This UPD phenotype manifests the imprinting effect, unmasked recessives, and possible mosaicism [12].

Few co-occurrence cases of mosaic whole-genome UPD have been reported in prenatal periods [13]. The fetal intrauterine phenotypes attainable are limited in ultrasonography, and some are invisible. Yi Zhang found only 50% of chromosomal mosaicism fetuses had ultrasound anomalies and seemed variable [14]. Therefore, it is a great challenge to evaluate the clinical outcome of these fetuses with mosaic chromosomal aneuploidy and UPD [13].

We have encountered several cases of mosaic chromosome aneuploidy and UPD in prenatal diagnosis. In this paper, we illustrate four examples of the prenatal diagnosis process and the clinical outcome of fetuses in our clinical work, hoping to provide references for future work.

## Methods

Four pregnant women, aged from 21 to 38 years with a gestational age of 18 + 5 to 23 + 4 weeks, were treated for prenatal diagnosis by amniocentesis because of a high risk of NIPT/serological screening or advanced age or fetal abnormal phenotype by ultrasound. The amniotic fluid and peripheral blood were collected from four pregnant women. The fetal skin, muscle, and kidney tissues in case 3 were also sampled after induced labor.

10 mL amniotic fluid was cultured, harvested, slides-prepared, and G-banded with conventional methods. True mosaicism was diagnosed by multiple aneuploid cells from at least two primary cultures and by analyzing more than 50 metaphase cells. Genomic DNA was extracted from amniocytes, maternal blood, and fetal tissues using the QIAamp DNA Blood/Tissue Kit (Qiagen, Germany). 10ng DNA of amniocytes and maternal blood were amplified separately by Goldeneye DNA Identification System 20 A (Beijing Jidian Corp.), and an extra amplification of case 4 proceeded by Specific Chr20 Kit (Shanghai Jingzhun Corp.). The PCR product was electrophoresed with ABI 3500Dx, and the Short tandem repeats(STR) data were analyzed by GeneMapper 5.0 software. The maternal cell contamination (MCC) and genetic origins of abnormal chromosomes were identified by comparing the fetal and maternal STRs. Chromosomal microarray analysis (CMA) proceeded with Affymetrix CytoScan^®^750 K Array (Afymetrix Inc, CA, USA) and Chromosome Analysis Suite Software (ChAS; version 4.0) amniocytes of case 1, 2, and 3, and the skin, muscle, and kidney tissues of the induced fetus, respectively. SurePrint G3^®^ 180 K chip(Agilent Inc, CA, USA) and Agilent CytoGenomics software were adopted in case 4. The methylation level of the amniocytes in case 3 was analyzed with Probemix ME028 for Prader-Willi syndrome (PWS)/Angelman syndrome (AS) and Probemix ME030 for Beckwith-Wiedemann syndrome (BWS)/Russel-Silver syndrome (RSS), respectively. The results were analyzed by Coffalyser.net software (MRC-Holland). 10ng Genomic DNA of uncultured and cultured amniocytes of case 4 was fragmented, and a library was prepared using the PCR-free method (Beijing Annoroud Biotechnology Co., Ltd.). The CNV-seq results were analyzed using the “Annorcloud” platform after being sequenced on the Nextseq 550AR (Illumina, Inc.). The exome sequencing library of case 1 was constructed from the NimbleGen SeqCap EZ MedExome kit and sequenced by a 2 × 150 bp double-ended method on an Illumina Novaseq 6000 high-throughput sequencer. The sequencing data were analyzed with Polyphen-2, SIFT, and Mutation Taster software.

## Results

The amniocyte karyotypes of case 1 was 46,XX[50]; case 2 was 47,XX,+15[3]/46,XX[125]; case 3 was 45,X[9]/46,XX[70]; and case 4 was 47,XY,+20[45]/46,XY[5] (not shown).

**Fig. 1:**
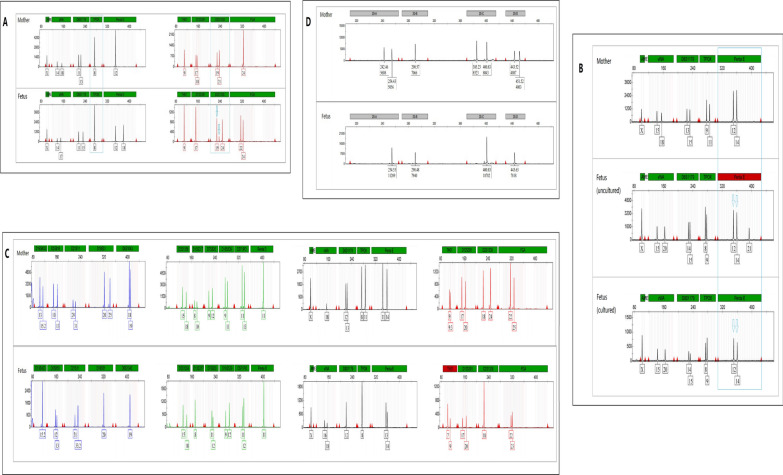
The STR results of case 1–4. None of the STR loci of the amniocytes in four cases showed MCC. **A** In fetus 1, the chr2 had two STR loci. TPOX was a single peak with no information, and D2S1338 had three fluorescence peaks (19/21/24) with areas of 5246/1279/5020, and two (19 and 21, indicated by the blue arrows) were the same as the mothers’. **B** In case 2, the STRs of chr15 in uncultured amniocytes had three fluorescence peaks (12/14/21 with areas ratio of 1: 1: 0.4), and two (12 and 14, indicated by the blue arrows) were the same as the mothers’. The STRs of cultured amniocytes had two fluorescence peaks (12/14) with an area ratio of 1.2. **C** The STR showed a bias in area ratios of multiple bimodal peaks with higher paternal in case 3. The alleles ratios of paternal and maternal were about 1.3–1.5 and excluded the possibility of paternal heterodisomy. **D** In fetus 4, the four single-peak STRs of chr20 were identical to one of her mother’s alleles, indicating her maternal origin

**Fig. 2:**
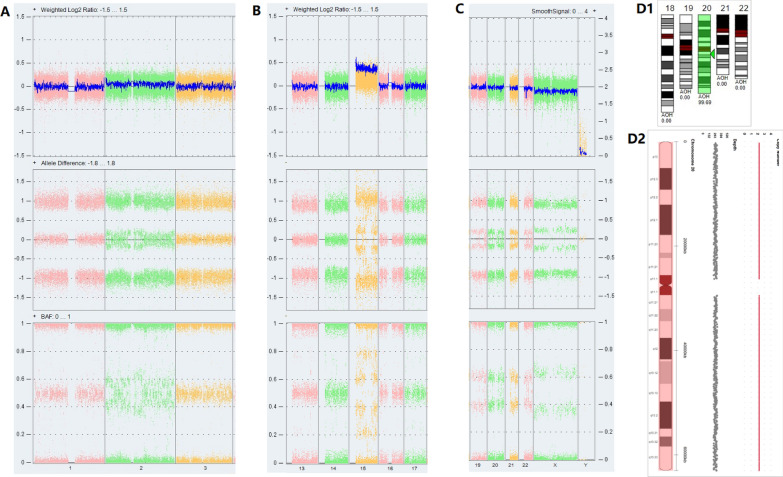
The mosaic chromosomal aneuploidy and uniparental disomy results of CMA/CNV-seq in fetal amniocytes. **A** The chip results of case 1 showed that the chr2 increased by about 7%, but the BAF and AD lines showed a higher amount of chr2, indicating co-existed UPD(2) and T2. By calculating, UPD(2) was about 12%. **B** The chr15 of case 2 increased by about 60%, and the BAF and AD showed several probes signal bands with locus regions of homozygosity (ROH). **C** The chrX of case 3 was significantly less than the autosome(about 13%). However, the actual levels of BAF and AD disagreed with the X chromosome mosaic, indicating additional UPD(X)(about 20%). In case 4, there was no abnormal CNV in uncultured amniocytes on the chip besides isoUPD of chromosome 20 (**D1**). However, the results of CNV-seq of the uncultured and cultured amniocytes presented none and 20% T20, respectively (**D2**)

Analyzed by CMA/CNVseq combined with STR, the results of amniocyte showed four cases were all mosaic UPD and chromosomal aneuploidy, as shown in Figs. 1 and 2, and Table 1. Case 1 was 7% T2 and 12% UPD(2)mat. Case 2 was 60% T15 and 40% UPD(15)mat. Case 3 was 13% 45,X and 20% paternal genome-wide UPD. Case 4 was UPD(20)mat. None of the T20 cells were detected in uncultured amniocytes by CMA and CNV-seq, but 20% in cultured amniocytes by CNV-seq.

**Fig. 3:**
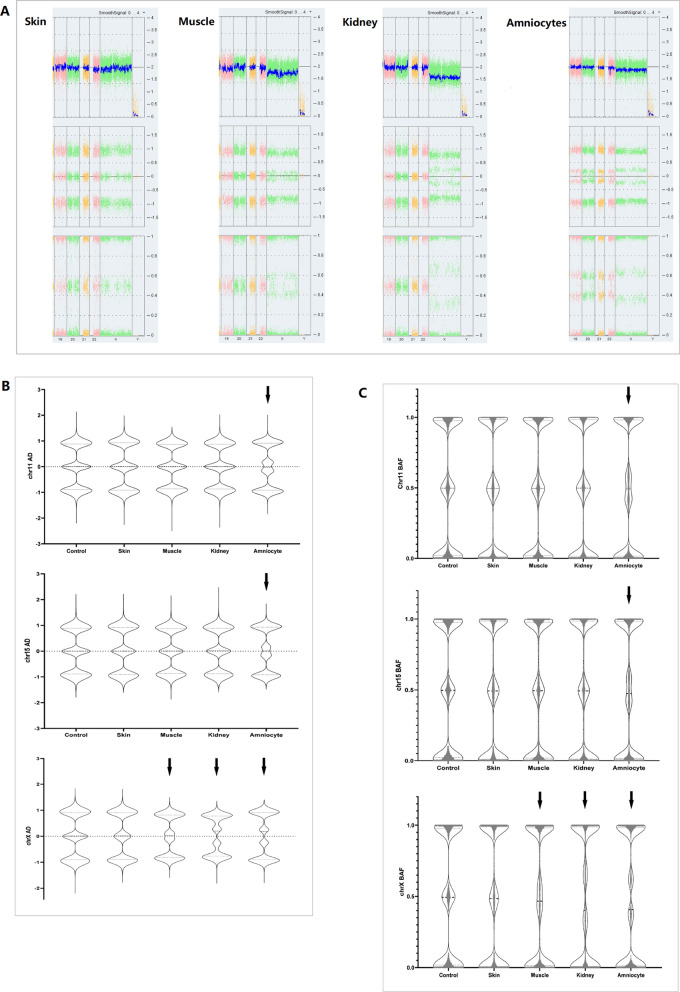
Comparison of chromosome mosaic levels between amniocytes, skin, muscle, and kidney in fetus 3. The chip result (**A**) showed a decrease of chrX in amniocytes, muscles, and kidneys except for skin, and the mosaic levels of 45,X were calculated to be 13%, 25%, and 34%, respectively. The AD and BAF files of the chr11, chr15, and chrX were analyzed comparatively among amniocytes, skin, muscles, and kidneys (**B**, **C**). The results showed that chr11, chr15, and chrX of amniocytes were abnormal, while the muscles and kidneys only had chrX abnormalities, and levels of chrX were different, as shown by the arrow in Fig. 3

No pathogenic or likely pathogenic variants of SNV/Indel were found in case 2. In fetus 3, the chrX was disomy in the skin but decreased in amniocytes, muscle, and kidney. The mosaic levels of 45,X cells were calculated to be 13%, 25%, and 34%, respectively, as shown in Fig. 3. Although the MS-MLPS results of 11p15.5 and 15q11-13 regions did not show abnormalities, the mosaic UPD(11) and UPD(15) of amniocytes were deduced from the abnormal AD and BAF and normal smoothsignal in chip results.

### Clinical information, experimental results, and clinical outcomes of four cases with mosaic chromosomal aneuploidy and UPD

Table 1 listed the information on four cases, including clinical information and prenatal diagnostic indication, sample type, experimental results (karyotypes, STR, CMA, CNV-seq, MS-MLPA, WES), intrauterine phenotypes, and clinical outcomes.

**Table 1:** Clinical information, experimental results, and clinical outcomes of four cases with mosaic chromosomal aneuploidy and UPD

Case no.	Clinical information and prenatal diagnostic indication	Sample type	Experimental results						Fetal phenotypes	Clinical outcomes
			Karyotypes	STR	CMA	CNVseq	MS-MLPA	WES		
1	A 33-year-old pregnant woman whose fetus had a normal phenotype on ultrasound. Due to a high-risk result for chromosome 2 on cell-free non-invasive prenatal test and reversed alpha wave in the ductus venosus and amniocentesis was performed at nineteen weeks and three days of gestation	Amniocyte	46,XX	The TPOX locus of the fetus in chr2 was a single peak without information, and the D2S1338 locus had three fluorescence peaks (19, 21, and 24 with an area of 5246, 1279, and 5020), and two of them (19 and 21) were the identical sizes with the mothers (Fig. 1A)	The amniocyte microarray results of case 1 showed that chromosome 2 was increased by about 7%. The B Allele Frequency (BAF) indicated the chr2 was a UPD, which consisted of isoUPD and hetUPD, and the proportion of UPD(2) was about 12% (Fig. 2A)	ND	ND	None of pathogenic or likely pathogenic snv/Indel variants were detected	The venous catheter wave was reversed. The fetus had no significant abnormal phenotype and was normal in size	The pregnancy was continued, and the baby was delivered to term. The baby’s growth and development were not abnormal after the follow-up of half a year
2	A 38-year-old pregnant woman. Due to a high-risk result for chromosome 15 on cell-free non-invasive prenatal test and advanced-age and amniocentesis was performed at gestational age 18 + 5	Amniocyte	47,XX,+15[3]/46,XX[125]	The Penta E locus of the uncultured amniocytes in chr15 had three peaks (12, 14, 21 with an area ratio of 1:1:0.4), and two of them (12 and 14) were the identical sizes with the mothers’. The peaks of cultured amniocytes were 12 and 14, with a ratio of 1.2 (Fig. 1B)	The microarray results of the uncultured amniocytes suggested that there was about a 60% increase of the chr15, and the BAF and AD showed several bands (Fig. 2B)	ND	ND	ND	The fetus had no significant abnormal phenotype by Ultrasound	The pregnancy was terminated
3	A 21-year-old pregnant woman. Ultrasound presented with fetal pericardial effusion of 3 mm and an adrenal cystic echo at twenty-three weeks. The amniocentesis proceeded at twenty-three weeks and four days	Amniocyte, fetal tissues (skin, muscles, and kidneys)	45,X[9]/46,XX[70]	Paternal alleles of amniocytes were significantly more than maternal ones because their fluorescence peak ratios were about 1.3–1.5, and paternal heterodisomy was excluded (Fig. 1C)	The microarray results of the amniocytes were arr(X) x1-2, mos UPD(1–22, X). The chrX was less (45,X accounted for about 13%) and mosaic whole genome UPD (about 20%)(Fig. 2C). The chip result (A) showed a decrease of chrX in amniocytes, muscles, and kidneys except for skin. And the mosaic levels of 45,X cells were calculated to be 13%, 25%, and 34%, respectively (Fig. 3)	ND	The MS-MLPA results of 11p15.5 and 15q11-13 regions in amniocytes showed normal	ND	Ultrasound showed the fetus had a pericardial effusion of 3 mm and an adrenal cystic echo at 23 weeks of gestation	The pregnancy was terminated
4	A 35-year-old pregnant woman with a high risk of trisomy 21, due to elevated beta-HCG MOM of 3.24 (reference value is below 2.5) in mid-term pregnancy serum screening. The amniocentesis proceeded at a gestational age of twenty weeks and two days	Amniocyte	47,XY,+20[45]/46,XY[5]	The four single-peak STR of chr20 in the amniocytes (Fig. 1D) were identical to one of the mothers’, indicating their maternal origin.	The CMA results of the uncultured amniocytes showed no copy number variation (CNV) besides isoUPD of chr20 (Fig. 2D1)	Uncultured amniocytes: normal. Cultured amniocytes: 20% T20 was found (Fig. 2D2)	ND	ND	Ultrasound indicated intrauterine growth restriction after 22 weeks	The pregnancy was terminated

*ND* No detected

## Discussion

The effects of mosaic chromosomal aneuploidy and UPD on fetuses are related to different chromosomes, mosaic levels, and mosaic tissues, which affect the intrauterine phenotype and clinical outcome. The four fetuses in this study had low-level mosaic chromosomes of T2, T15, monosomy X, and T20, respectively. The clinical influence of UPD is directly related to the gene content and size of the affected chromosomal region. If imprinted genes were involved, UPD could cause imprinted diseases, such as Beckwith-Wiedemann syndrome (BWS, patUPD11), Kagami-ogata syndrome (KOS, patUPD14), Angelman syndrome (AS, patUPD15)/PWS (PWS, matUPD15), pseudohypoparathyroidism (patUPD20) /Mulchandani-Bhoj-Conlin syndrome (MBCS, matUPD20) [15]. In addition, UPD events also increase the risk of recessive genetic disorders. The UPD of this study involved chr2, chr15, chr20, and the whole genome. In utero, fetuses usually have less visible phenotypes. However, when the prenatal imaging of the fetus is consistent with typical UPD manifestations of the KOS and BWS, chromosomal or genetic abnormalities should be validated by laboratory methods [16].

**Fig. 4:**
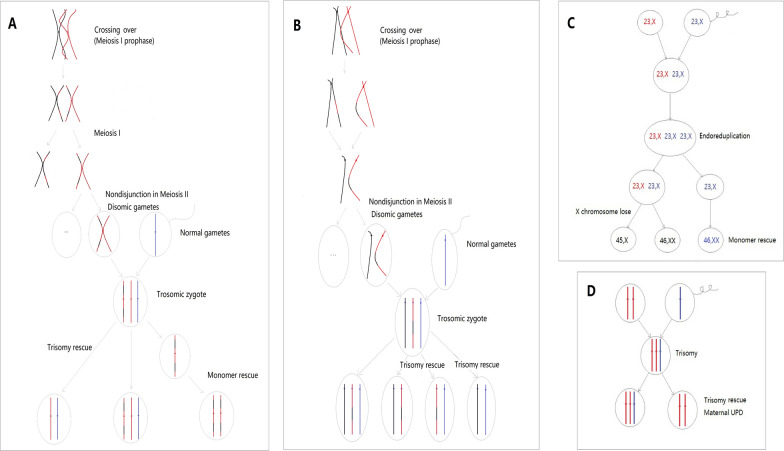
Formation mechanisms of mosaic aneuploidy and UPD in four cases. **A** In case 1, the maternal chr2 was crossed over in meiosis I prophase, and then, multiple adverse events, such as non-separation of the second meiosis, trisomy rescue, and monomeric self-rescue, occurred continuously on chr2 during meiosis and mitosis Chr2 consisted of normal cell, trisomy, and matUPD disomy cells. **B** In case 2, the maternal chr15 were homologous recombination in meiosis I prophase, then, non-separation of the meiosis II, trisomy rescue of the trisomic zygote happened on chr15 during meiosis and mitosis. Chr15 consisted of normal cells, trisomy, and matUPD disomy cells. **C** Three adverse events occurred successively in case 3, including endoreduplication and monomer rescue of the paternal genome and loss of a chrX in some cells during the anaphase of mitosis. Thus, a mosaic consisted of paternal whole genome UPD, biparental 46,XX, and 45,X cells. **D** For case 4, two adverse events occurred in succession, including no separation in the MII phase of the ovum and trisomy self-rescue of chr20. However, very few trisomy cells remained after an incomplete self-rescue. Thus, the mosaic compositions in uncultured amniocytes were maternal UPD(20) cells (> 90%) and T20 cells (< 10%), estimated by the detection limit of the CMA and CNV-seq

In our four cases, we found more than two adverse genetic events happened in succession during their meiosis and mitosis as in previous reports [17] and proposed a possible formation mechanism of mosaic chromosomal aneuploidy and UPD. See Fig. 4 for details.

We speculate multiple events might occur continuously on chromosome 2, including homologous recombination in prophase I, no separation in meiosis II, and rescue of trisomy and monosomy during mitosis. The chr2 consisted of normal cells, trisomy, and maternal UPD cells. Blaicher et al. reported that a complete T2 was associated with acardiac fetuses and poor clinical outcomes [18], and mosaic T2 has variable phenotypes, such as nonspecific phenotypes, intrauterine growth restriction, intrauterine fetal death, or stillbirth. Trisomy 2 is almost always confined to the placenta [19]. T2 mosaic in a fetus is a rare high-risk of abnormalities [20]. UPD(2) is one of the relatively rare UPD chromosomes. There were reports that children of UPD(2) were normal after birth [21], and there were also reports of patients with multiple recessive diseases caused by the homozygous state of multiple mutated recessive genes due to UPD(2). Chr2 consisted of heterodisomy and isodisomy, hinting at a higher risk of recessive allelic homozygous [22, 23]. Although any pathogenic or likely pathogenic variant was excluded through WES, the clinical outcome still could not be accurately predicted because of the uncertain mosaic levels of T2 cells in different tissues. There were no abnormalities in three subsequent ultrasound examinations, and the size of the fetus was consistent with that of gestational age. After being informed fully, the pregnant woman chose to continue the pregnancy and gave birth to a normal full-term baby. The follow-up showed no abnormality in the baby’s growth and development until one year after birth.

Case 2 was a 60% mosaic T15 in the uncultured amniocytes with CMA, but only low-level trisomic mosaic(3 out of 128 metaphases) in cultured amniocytes, which may be explained by the selective growth of cultured cells [24, 25]. Meanwhile, UPD(15)mat consisted of heterodisomy and isodisomy. We speculated on the formation progress, including the crossing over in meiosis I prophase and nondisjunction in meiosis II, a part of trisomic cells rescue successfully from trisomic zygote. The chr15 contained the well-known imprinted genes, and the duplication of chr15 has a definite clinical phenotype after birth, although the fetal ultrasound examination found no obvious abnormality at twenty-one weeks and five days. The pregnant woman chose to terminate the pregnancy at 25 weeks after being fully informed.

Case 3 was a mosaic genome-wide paternal UPD and mosaic 45,X. We report for the first time that a fetal mosaic whole-genome UPD with mosaic monosomy X. It may be formed by multiple adverse events such as endoreduplication of the paternal genome, loss of an X chromosome during mitosis, and self-rescue of paternal chromosome monomeric cells. Although the abnormal hints of AD and BAF signal in amniocytes, no significant methylation abnormalities on chromosomes 11 and 15 were found by the MS-MLPA method, which may result from the low-level mosaic of UPD(11) and UPD(15) beyond the detection limit. The results of multiple points in the MS-MLPA diagram were lower than 0.5, suggesting a low methylation level, similar to the mosaic methylation reported by Aypar [26] and Morandi [27]. Nazarian et al. concluded that patients with mosaic imprinting defects had mild or atypical AS [28]. Buiting et al. found that patients with methylated mosaic had an extensive clinical spectrum, ranging from typical AS to mild AS to atypical AS [29]. Wey et al. emphasized that it is difficult to determine the severity and phenotypic expression profile based on the ratio of normal to abnormal cells [30]. The individuals with mosaic whole-genome UPD could survive to adulthood [31]. The clinical symptoms of postnatal patients with mosaic whole-genome UPD varied to the mosaic levels and tissues [13, 32]. Some of them had tumors on the adrenal and other tissues [33, 34]. Comprehensively analyzing the low-level methylation and ultrasound manifestations of the adrenal cyst and pericardial effusion, the clinical outcome of the fetus has been evaluated as disadvantageous. The pregnant woman eventually chose to terminate the pregnancy after being fully informed.

In case 4, T20 cells were detected by karyotype and CNVseq methods in cultured rather than uncultured amniocytes, which may be explained by the selective growth of trisomy cells in the cultivation process [24, 25] and the limited detection ability of CMA and CNVseq for low-level mosaic [35]. The mosaic condition of this fetus mainly resulted from incomplete trisomy self-rescue, and most of the diploid cells successfully self-rescued were maternal UPD(20). T20 is one of the more common mosaic trisomies detected in amniocentesis, with a benign outcome in over 90% of reported cases [36]. Fetuses with a proportion of 14–100% T20 cells had favorable clinical outcomes [37, 38]. However, our fetus had an additional rare matUPD(20) associated with fetal intrauterine dysplasia, which involved imprinting genes GNAS, and its clinical manifestations are severe feeding difficulties, stunted growth, preterm birth, and intrauterine/postpartum growth retardation [39, 40]. The fetal ultrasound examination found no obvious abnormality at twenty weeks and two days. However, the fetal intrauterine growth restriction became increasingly apparent in the following three weeks after 22 gestational weeks, indicating the outcome was adverse. The pregnant woman was fully informed and chose to terminate the pregnancy at 25 weeks.

UPD and chromosomal aneuploidy often co-occur, so it is necessary to analyze them simultaneously when chromosomes with imprinted regions are involved or known carriers of a recessive allele. Because SNP-based chips could not detect heterodisomy, other methods, such as STR or trio-WES, should be supplemented when mixed iso/heterodisomy is suspected. Moreover, the detection abilities of qualitative MS-MLPA, SNP array, and microsatellite for low-level mosaics are limited, with a sensitivity of about 10% [31], so quantitative methylation analysis is recommended to compensate [41]. Genetic counseling for such fetuses is crucial, and doctors should fully inform pregnant women so that they can make informed choices. The information includes but is not limited to these contents: the affected chromosomes, mosaic levels, mosaic tissues, methylation status, recessive gene variations on the UPD chromosome, intrauterine phenotypes, clinical manifestations of similar patients after birth, and current clinical treatment available [42].

## Data Availability

The datasets used and/or analysed during the current study are available from the corresponding author on reasonable request.
